# Supplementary material for: High-Throughput In Vitro Screening Identified Nemadipine as a Novel Suppressor of Embryo Implantation
Source: Int J Mol Sci. 2022 May 3;23(9):5073. doi: 10.3390/ijms23095073 (PMC9103851; doi:10.3390/ijms23095073)
Supplement: Supplementary file 1 [file ijms-23-05073-s001.zip › ijms-1699416-sup.pptx]

## Slide 1
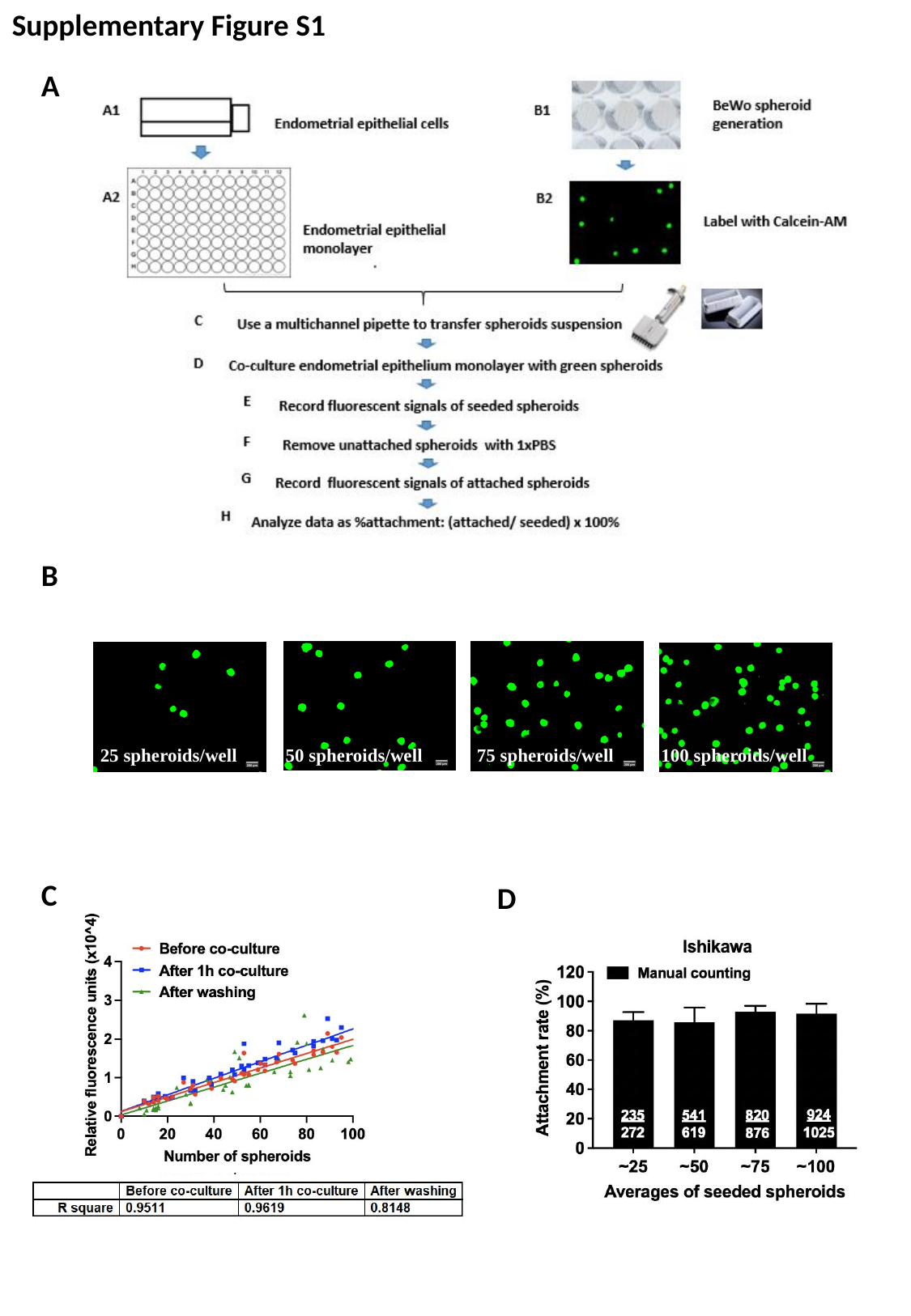

Supplementary Figure S1
A
B
25 spheroids/well
50 spheroids/well
75 spheroids/well
100 spheroids/well
C
D

## Slide 2
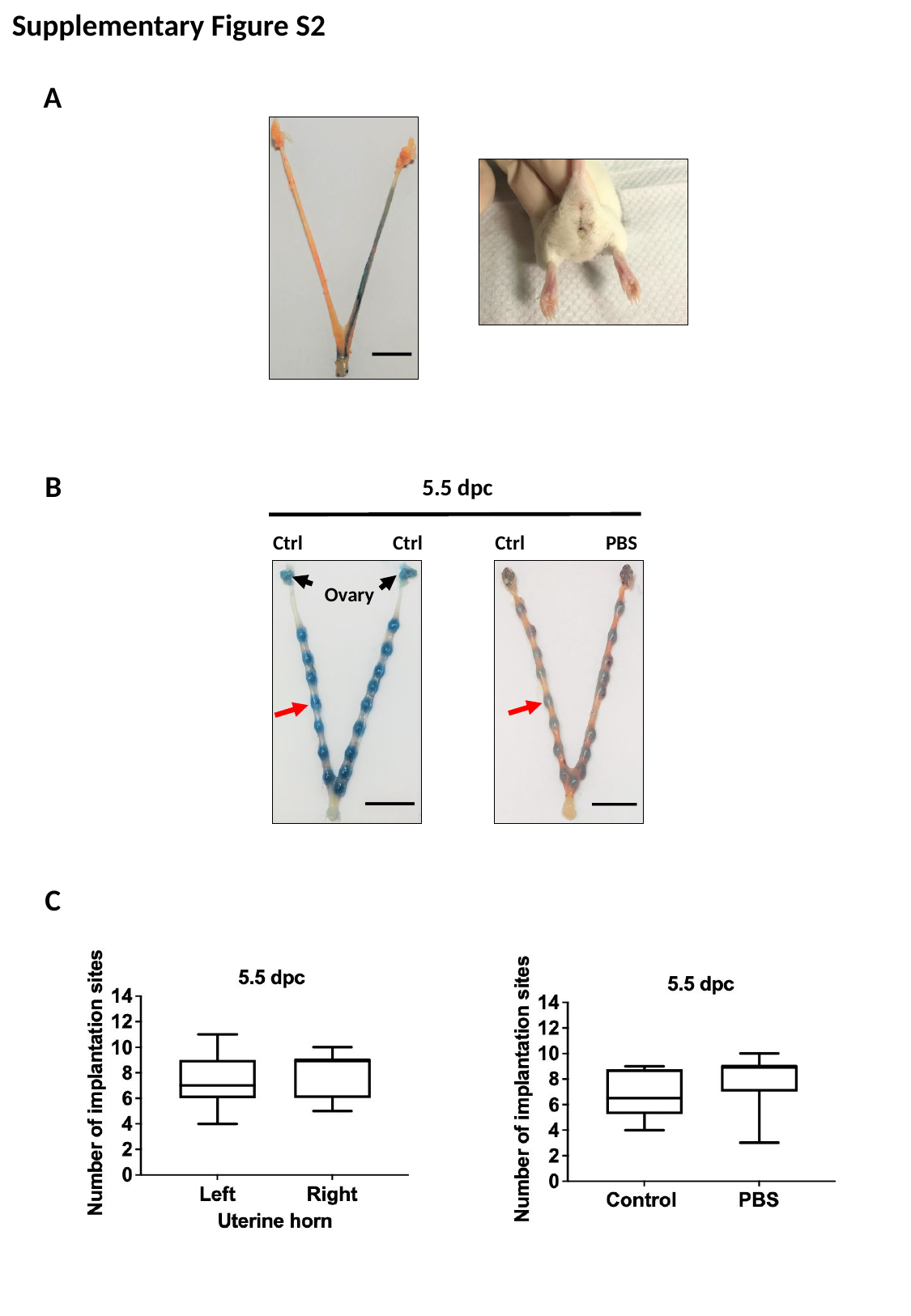

Supplementary Figure S2
A
B
5.5 dpc
Ctrl
Ctrl
Ctrl
PBS
Ovary
C

## Slide 3
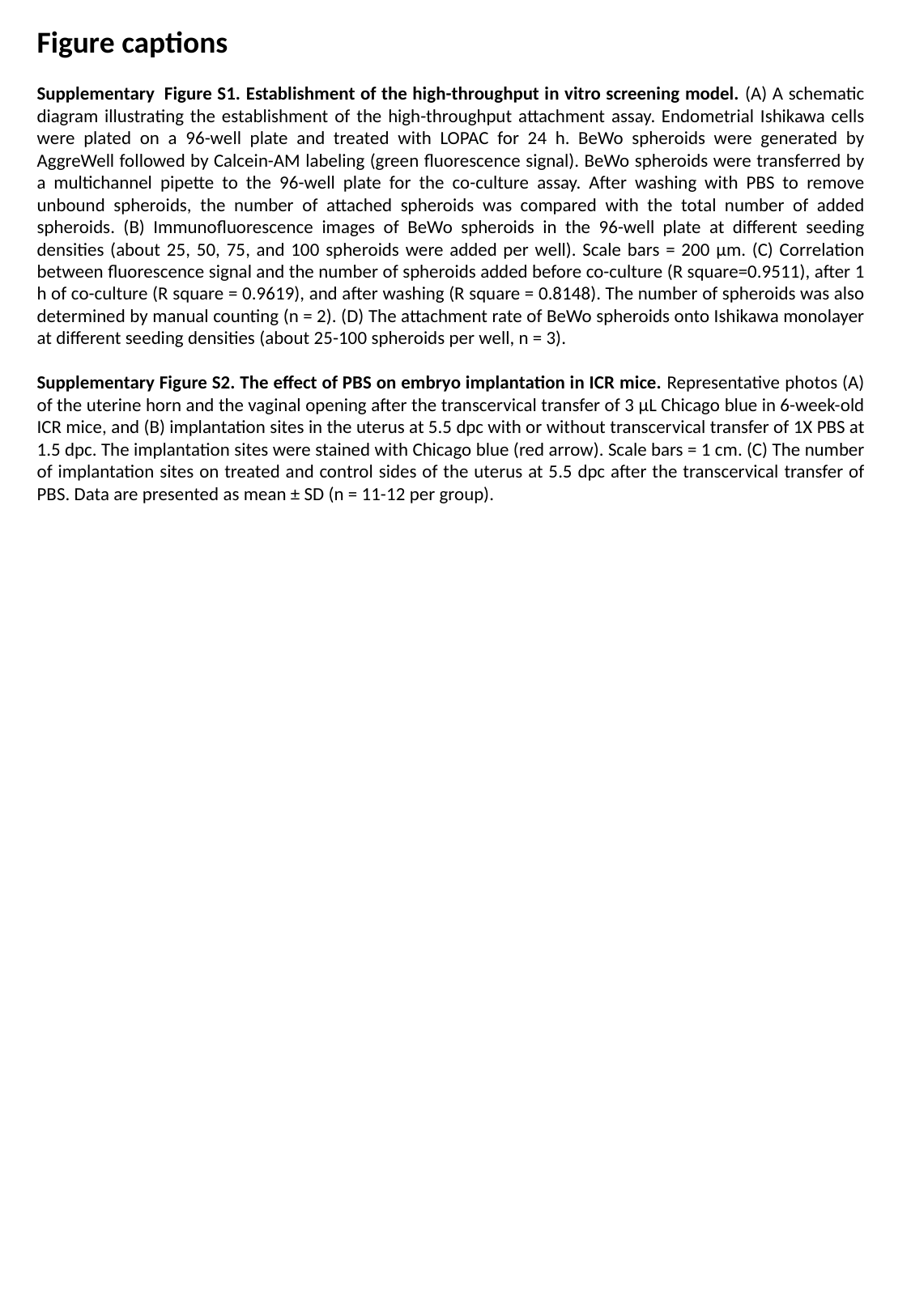

Figure captions
Supplementary  Figure S1. Establishment of the high-throughput in vitro screening model. (A) A schematic diagram illustrating the establishment of the high-throughput attachment assay. Endometrial Ishikawa cells were plated on a 96-well plate and treated with LOPAC for 24 h. BeWo spheroids were generated by AggreWell followed by Calcein-AM labeling (green fluorescence signal). BeWo spheroids were transferred by a multichannel pipette to the 96-well plate for the co-culture assay. After washing with PBS to remove unbound spheroids, the number of attached spheroids was compared with the total number of added spheroids. (B) Immunofluorescence images of BeWo spheroids in the 96-well plate at different seeding densities (about 25, 50, 75, and 100 spheroids were added per well). Scale bars = 200 μm. (C) Correlation between fluorescence signal and the number of spheroids added before co-culture (R square=0.9511), after 1 h of co-culture (R square = 0.9619), and after washing (R square = 0.8148). The number of spheroids was also determined by manual counting (n = 2). (D) The attachment rate of BeWo spheroids onto Ishikawa monolayer at different seeding densities (about 25-100 spheroids per well, n = 3).
Supplementary Figure S2. The effect of PBS on embryo implantation in ICR mice. Representative photos (A) of the uterine horn and the vaginal opening after the transcervical transfer of 3 μL Chicago blue in 6-week-old ICR mice, and (B) implantation sites in the uterus at 5.5 dpc with or without transcervical transfer of 1X PBS at 1.5 dpc. The implantation sites were stained with Chicago blue (red arrow). Scale bars = 1 cm. (C) The number of implantation sites on treated and control sides of the uterus at 5.5 dpc after the transcervical transfer of PBS. Data are presented as mean ± SD (n = 11-12 per group).
